# A dual-fMRI investigation of the iterated Ultimatum Game reveals that reciprocal behaviour is associated with neural alignment

**DOI:** 10.1038/s41598-018-29233-9

**Published:** 2018-07-18

**Authors:** Daniel J. Shaw, Kristína Czekóová, Rostislav Staněk, Radek Mareček, Tomáš Urbánek, Jiří Špalek, Lenka Kopečková, Jan Řezáč, Milan Brázdil

**Affiliations:** 1 Department of Psychology, Aston University, Birmingham, B4 7ET United Kingdom; 2 CEITEC – Central European Institute of Technology, Masaryk University, Brno, Czech Republic; 3 Department of Economics, Faculty of Economics and Administration, Masaryk University, Brno, Czech Republic; 4 Department of Public Economics, Faculty of Economics and Administration, Masaryk University, Brno, Czech Republic; 5 Institute of Psychology, Academy of Sciences of the Czech Republic, Brno, Czech Republic

## Abstract

Dyadic interactions often involve a dynamic process of mutual reciprocity; to steer a series of exchanges towards a desired outcome, both interactants must adapt their own behaviour according to that of their interaction partner. Understanding the brain processes behind such bidirectional reciprocity is therefore central to social neuroscience, but this requires measurement of both individuals’ brains during real-world exchanges. We achieved this by performing functional magnetic resonance imaging (fMRI) on pairs of male individuals simultaneously while they interacted in a modified iterated Ultimatum Game (iUG). In this modification, both players could express their intent and maximise their own monetary gain by reciprocating their partner’s behaviour – they could promote generosity through cooperation and/or discourage unfair play with retaliation. By developing a novel model of reciprocity adapted from behavioural economics, we then show that each player’s choices can be predicted accurately by estimating expected utility (EU) not only in terms of immediate payoff, but also as a reaction to their opponent’s prior behaviour. Finally, for the first time we reveal that brain signals implicated in social decision making are modulated by these estimates of EU, and become correlated more strongly between interacting players who reciprocate one another.

## Introduction

Many social interactions comprise a series of repeated exchanges between individuals. Within the constraints of social convention, such dynamic contexts demand a process of mutual reciprocity^1^; over the course of a repeated dyadic exchange, for example, both interactants modify their own behaviour in response to their partner’s in an attempt to steer the interaction towards a desired outcome. In this light, repeated exchanges unfold as a two-in-one process whereby each individual’s behaviour is simultaneously a consequence of and antecedent to that of their partner’s. Advancing our understanding of the brain processes underlying such reciprocity is therefore central to social neuroscience research^2^, but this requires measurement of both interactants’ brains whilst they engage in naturalistic social exchanges.

The Ultimatum Game (UG^3^) presents a simple paradigm to investigate dyadic interaction. A Proposer is asked to choose from a range of options how they wish to divide a sum of money (the “pie”) between themselves and a Responder. The Responder then chooses whether to accept or reject the offer; if they accept it then the pie is divided accordingly, but if they reject it then neither player receives any payoff. Contrary to game theoretic predictions of rational behaviour, modal offers are around 40% of the pie and Responders reject proposals of 20% approximately half the time^4^. Responders’ behaviour appears to reflect an aversion to inequity; they consider it unfair to be offered disproportionately less than their interaction partner (disadvantageous inequity), and reject such proposals as a challenge to subjugation^5^. Consistent with this notion, neuroimaging studies demonstrate that rejected offers elicit neural responses in brain systems implicated in subjective feeling states such as pain and disgust (anterior insula [AI]^6^) and social information processing (anterior [ACC] and anterior-mid cingulate cortices [aMCC; see^7^]; for meta-analytic reviews see^8,9^). In contrast, Proposers’ offers are believed to reflect strategic behaviour; in an attempt to maximise their own payoff they avoid offers that are likely to be rejected, such as those with which they earn disproportionately more (advantageous inequity). Although fewer neuroimaging studies have investigated Proposers, the available evidence points to neural responses in frontal midline brain regions during offers that reflect such egoistic strategies (e.g.,^10–12^).

The UG is performed typically in a one-shot manner, however – a single round played, ending after the Responder accepts or rejects a proposed division. Whilst this simulates one-off social interactions, it fails to capture the bidirectional and reciprocal property of repeated exchanges^13^; in such contexts, we do not simply react to another’s behaviour but we *interact* with them in an attempt to bring about a desirable outcome. Furthermore, previous studies have examined UG performance between individuals who are anonymous to one another, thereby removing the social context in which the majority of day-to-day interactions take place and limiting the applicability of resulting behaviours. Investigating the sequential and reciprocal nature of real-world dyadic interactions requires an iterated UG (iUG^13–15^) in which multiple exchanges occur between the same players. In this situation, both players can adapt to the behaviour of their opponent in order to maximise their own payoff over recursive rounds; Responders can encourage equitable offers by rejecting disadvantageous ones, and Proposers can increase acceptance by adapting to Responder behaviour^16^. Alternatively, either player can adopt an unwavering strategy; by offering or accepting only those proposals that benefit themselves maximally, players can force their partner into a compromise over fairness and ultimate payoff. In other words, both players can express varying degrees of reciprocity over multiple rounds with the same partner.

Reciprocity unfolds as an indirect chain of neural events; through neural coupling, one individual’s brain activity results in a behavioural output, which then elicits systematic neural responses in their interaction partner to initiate a behavioural reaction^17^. As such, only by measuring brain signals in both interactants simultaneously can we begin to elucidate the interpersonal neural processes underlying reciprocity^18^. By employing this “hyperscanning” method, neuroscientific studies report spatially and temporally synchronised brain signals between interactants that vary with the nature of the social exchange (for reviews see^19,20^). One particular form of neural coupling is *alignment* – that is, correlated neural signals between brains^21^. This is analogous to a wireless communication system, through which a sender and receiver become synchronised to a transmitted signal (e.g., light or sound^22^). The signals driving neural alignment vary in their level of abstraction, however; while sensory cortices will align to interactants’ physical movements, correlated signals in higher-order brain regions reflect a shared understanding of the intentions behind the actions^18,21,23^.

To investigate whether reciprocity during real-world, repeated dyadic exchanges elicits patterns of neural alignment, the present study performed functional magnetic resonance imaging on pairs of individuals simultaneously (dual-fMRI) while they played a modified iUG designed to encourage reciprocity. Unlike other hyperscanning methods (e.g., fNIRS), dual-fMRI affords direct localisation of inter-brain effects within cortical *and* subcortical regions. To capture behavioural reciprocity over multiple rounds of economic exchange, we developed a novel adaptation of a model from experimental economics^24^ that fits each player’s round-by-round behaviour (the proposed division or its acceptance/rejection) to an estimate of expected utility (EU) on a given exchange. Crucially, this estimate of EU considered not only the distribution of payoff between players, thereby incorporating any social preferences (inequity aversion), but also the extent to which their choices reflect a reaction to their partner’s prior behaviour; if player A considers B’s past behaviour to have been fair then they will perceive greater utility in increasing B’s relative payoff, but if A believes B’s past behaviour to have been unfair they will take pleasure in decreasing B’s payoff in favour of their own (positive and negative reciprocity, respectively). By combining functional neuroimaging data with estimates of EU from our reciprocity model, we were then able to investigate whether brain responses map onto utility evaluations influenced by an opponent’s prior behaviour – that is, the neural coupling associated with reciprocal behaviour. We also assessed neural alignment specifically by measuring covariance in brain signals between interacting players^21,25^, and investigated whether this is related to their expression of reciprocity. We hypothesised that greater reciprocity would be associated with stronger co-activation between interacting players’ brains in regions implicated in fairness evaluations and strategic behaviour on the UG – namely, AI and ACC/aMCC.

Interestingly, when selecting a division from two alternatives (the choice set), Proposers are more likely to offer the fairer of the two when the other option becomes more selfish^26^. Furthermore, even if the fairer of the two options rewards the Responder with disproportionately less than their opponent, they are more likely to accept it because they consider it justified^27,28^. This contextual effect appears to reflect each player’s consideration of their opponent’s motivation: As a means of risk aversion, Proposers avoid the most selfish option because it is more likely to be rejected, decreasing its utility; and Responders accept offers that reward them disproportionately less if the alternative division incurs a greater cost to the Proposer. Since these decisions will be influenced by an evaluation of the other player’s prior behaviour, thereby eliciting more reciprocity, our modification of the iUG permitted comparisons between two different contexts: On Proposer-Responder (PR) rounds, the choice set required Proposers to choose between a division that presented themselves with either advantageous or disadvantageous inequity (e.g., 60:40 *vs*. 40:60). Since the cost to the Proposer was far greater for the latter (very generous) division, both players were more likely to regard the former (selfish) option as justified^28,29^. Moreover, very generous offers on PR exchanges indicate that the Proposer perceived greater utility in increasing the Responder’s relative payoff at a cost to themselves, signalling a high degree of co-operative intent. In contrast, on Proposer-Proposer (PP) exchanges the Proposer had to choose between two divisions that differed only in magnitude of advantageous inequity (e.g., 60:40 *vs*. 70:30). Since the relative increase in cost to the Proposer by offering the least selfish division was reduced, a very selfish offer on PP exchanges indicated low co-operative intent. Thus, choices on PR and PP rounds were intended to elicit strong expressions of positive and negative reciprocity by both players. We predicted greater neural alignment during the former, in which more shared (co-operative) intentionality would be elicited.

## Results

We report the results of non-parametric statistical analyses if Kolmogorov–Smirnov tests revealed that normality was violated by at least one of the assessed variables. Values present means (±SD).

### Behaviour

All players made their choices (the selection of a division to offer, and the decision to accept or reject the proposed division) within the 4 second limit on all exchanges. Interestingly, however, response times (RTs) differed between players and conditions; a mixed-plot ANOVA, with the within-subject factor Condition (PR *vs*. PP) and between-subject factor Player (Proposer *vs*. Responder), revealed that RTs were greater for Proposers compared with Responders (2007.28 [±439.89] *vs*. 1106.99 [±429.72] ms; F[1,36] = 46.73, p < 0.001, ηp^2^ = 0.57) and on PR relative to PP exchanges (1641.71 [±637.81] *vs*. 1472.56 [±608.78] ms; F[1,36] = 13.43, p = 0.001, ηp^2^ = 0.27). There was no Player-by-Condition interaction (F[1,36] = 1.07, p = 0.308, ηp^2^ = 0.029). This revealed that (a) both players took longer to make decisions when faced with a choice between advantageous and disadvantageous inequity, but (b) Responders had already begun to evaluate the choice set before an offer was made.

**Figure 1:**
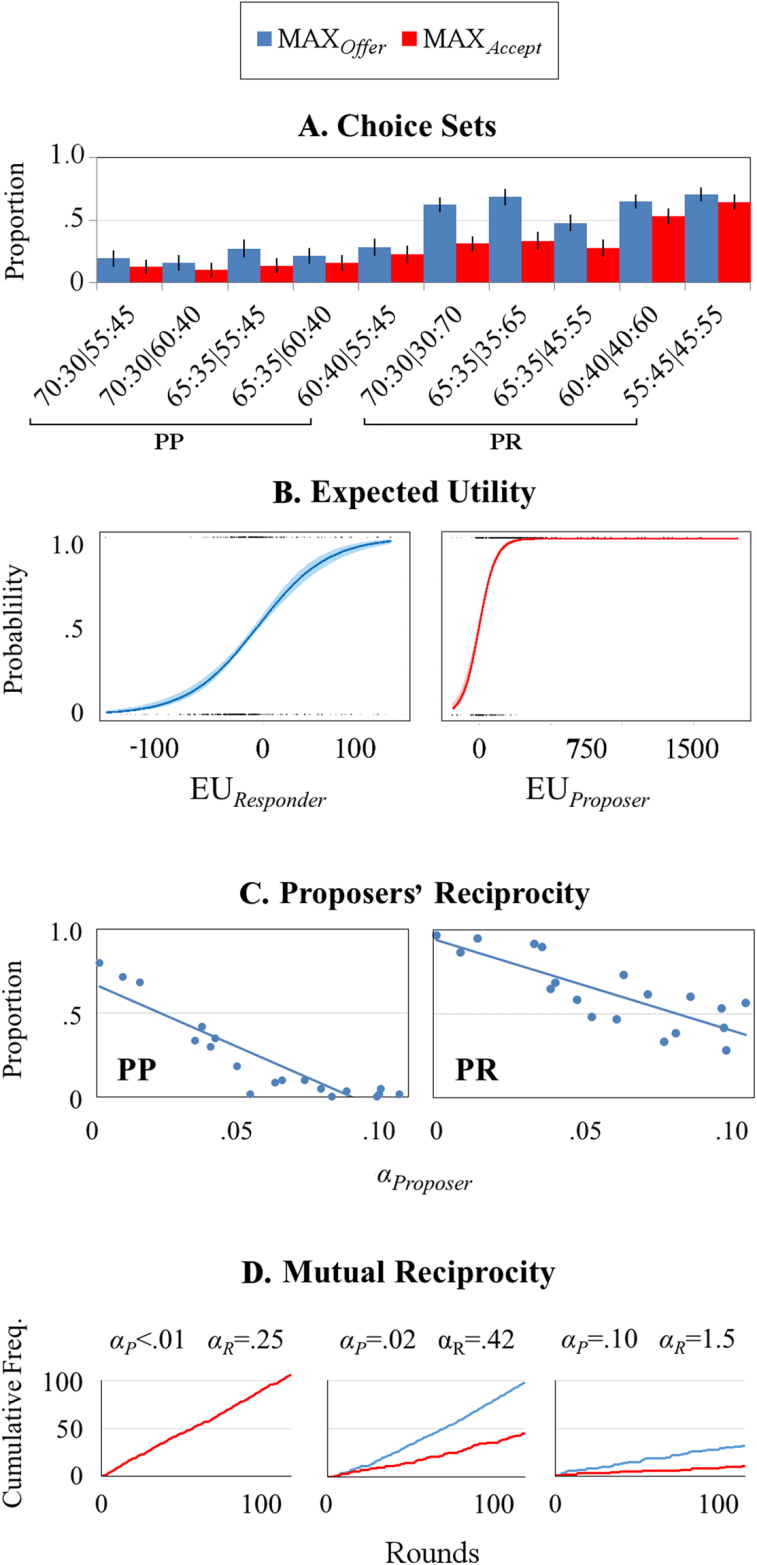
Patterns of player behaviour. (**A**) Mean (±SD) proportions of offers of maximal advantageous inequity (MAX_*Offers*_; blue) and their acceptance (MAX_*Accepts*_; red) across all dyads for each of the 10 choice sets. This indicates more MAX_*Offers*_ and MAX_*Accepts*_ across PR relative to PP rounds, which was confirmed with direct comparisons (see text). Error bars present standard deviations. (**B**) Probabilities of MAX_*Offers*_ and MAX_*Accepts*_ plotted as a logistic function of expected utility (EU), as estimated with the reciprocity model. (**C**) The proportion of MAX_*Offers*_ in each condition plotted as a function of Proposers’ reciprocity (*α*), as estimated by the reciprocity model. This reveals that with increasing reciprocity, Proposers were less likely to offer the division that benefited themselves maximally. (**D**) Cumulative frequencies of MAX_*Offers*_ and MAX_*Accepts*_ over all 120 UG rounds, for three example dyads. For the leftmost dyad, estimates of reciprocity were low for both the Proposer (*α*_*P*_) and Responder (*α*_*R*_). This is reflected in high number of MAX_*Offers*_ and MAX_*Accepts*_; the Proposer was free to offer divisions that benefited themselves maximally because the Responder did not challenge such proposals with rejections (negative reciprocity). In the middle dyad, the Responder did challenge MAX_*Offers*_ and this is reflected in a higher reciprocity estimate. These rejections did not sway the Proposer, however; they continued to propose such offers, reflected in a low reciprocity estimate. In the rightmost dyad, estimates of reciprocity were high for the Proposer and Responder. This is reflected in a low number of MAX_*Offers*_ and MAX_*Accepts*_; the Responder was unwilling to accept such offers, and the Proposer reacted to this with less advantageously inequitable proposals.

Next we assessed the pattern of proposals and decisions across choice sets, both within and between the PR and PP conditions. We focused on two aspects of player behaviour; specifically, the proportion of offers that benefited the Proposer maximally (i.e. those with maximal advantageous inequity; MAX_*Offer*_), and the proportion of these offers that were accepted by the Responder (MAX_*Accept*_). Figure 1A presents the distribution of each behavioural measure. This indicates higher proportions of both MAX_*Offer*_ and MAX_*Accept*_ for choice sets comprising the PR relative to the PP condition, which was confirmed with non-parametric comparisons (respectively, Z_[19]_ = 3.82, p < 0.001; and Z_[19]_ = 2.11, p = 0.033). The number of MAX_*Offer*_ and MAX_*Accept*_ also appeared to decrease with higher payoff for the Proposer or, in turn, lower payoff for the Responder. To quantify this, for each choice set we computed the payoff for each player presented by the division with maximal advantageous inequity. Spearman correlations confirmed that with increasing payoff for the Proposer, both MAX_*Offer*_ and MAX_*Accept*_ decreased (respectively, ρ_[18]_ = −0.53, p = 0.016; and ρ_[18]_ = −0.64, p = 0.003). Since Proposers selected an offer from two alternatives, the degree of payoff in the division with maximal advantageous inequity could also be expressed relative to the other option. To investigate whether MAX_*Offer*_ and MAX_*Accept*_ differed according to this relative measure, we compared the payoff to each player between the two divisions of each choice set; higher values represented a relative increase in the Proposer’s payoff or, in turn, a decrease in the Responder’s payoff for the division with maximal advantageous inequity (see Table S1). No significant relationships were observed between this relative measure of payoff and MAX_*Offer*_ or MAX_*Accept*_ (respectively, ρ_[18]_ = 0.39, p = 0.089; and ρ_[18]_ = 0.29, p = 0.217). Together, these results imply that neither player’s choices were driven solely by absolute or relative measures of payoff, which was confirmed in subsequent modelling procedures (see below).

We then applied our adapted reciprocity model to estimate the degree to which each player’s choices reflected reciprocal reactions to their partner’s prior behaviour, and from this we modelled each player’s round-by-round EU. For Proposers, greater values of EU represent higher utility for the division with least advantageous inequity (the generous offer); for Responders, it represented greater utility in accepting a proposed division. As shown in Fig. 1B, the probability of MAX_*Offers*_ and MAX_*Accepts*_ varied according to EU; indeed, estimates of EU correctly predicted these behaviours on 73.46 (±7.58 [AIC = 2345.5; BIC = 2454.4; Log-likelihood = −1153.7]) and 85.57 percent of rounds (±10.14 [AIC = 1272.2; BIC = 1381.1; Log-likelihood = −617.1]), respectively. Reciprocity parameters, *α*, were lower for Proposers (0.06 [±0.03]) than Responders (0.41 [±0.37]; Z_[18]_ = 5.04, P < 0.001), suggesting that the latter players’ decisions reflected stronger reactions to their partner’s offers. Estimated values of *α* for Proposers were correlated negatively with MAX_*Offers*_ across the PR (ρ_[17]_ = −0.78, p < 0.001) and PP condition (ρ_[17]_ = −0.88, p < 0.001), however; the more reciprocity they showed, the less likely there were to offer divisions that benefited themselves maximally (see Fig. 1C). No such relationship was observed between Responders’ *α* and MAX_*Accepts*_ for either the PR (ρ_[17]_ = −0.15, p = 0.551) or PP condition (ρ_[17]_ = −0.07, p = 0.786). Finally, Proposer *α* estimates correlated positively with the amount of time they took to decide the division they wished to offer on PR (ρ_[17]_ = 0.58, p = 0.009) but not PP rounds (ρ_[17]_ = −0.22, p = 0.377). Across all Proposers the optimal *Memory* parameter was 73, identifying the range of preceding rounds that maximized the accuracy of Proposers’ predictions of their opponents’ decisions. There was only a slight benefit beyond a range of 20, however; for round-by-round estimates of EU, estimates of each player’s reciprocity parameter, and the accuracy in predicting Proposers’ choices, correlations were highly similar for models with a *Memory* parameter of 20 and upwards (see Table S2).

To evaluate our adapted reciprocity model we compared it against a variety of alternatives (see Supplementary Materials for full model specifications). First we tested a nested model by fixing the reciprocity parameter to α = 0 for both players. This self-regarding model evaluated the assumption that both players care only about their own onetary payoff. The likelihood ratio (L) demonstrated that our reciprocity model outperformed this mself-regarding model when applied to both Proposers (L[19] = −1037.2 [AIC = 2074.5]; p < 0.001) and Responders (L[19] = −1553.0 [AIC = 3106.0]; p < 0.001). Second, given the relatively low estimates of Proposer’s reciprocity, we tested whether the reciprocity utility function should be applied only to Responders. We achieved this by evaluating the change in model fit by fixing only the Proposer’s reciprocity parameters to α = 0; the same reciprocity model was applied to Responders. Again, our model fitted Proposers’ choices more accurately than this nested self-regarding model (L[19] = −1609.3 [AIC = 3219.5]; p < 0.001). Finally, we assessed whether choices reflect learning processes over multiple rounds rather than reciprocal reactions. To do so, we modelled each player’s behavioural data with a three-parameter extension of the reinforcement learning model^30^. This model contains a forgetting parameter φ, an experimentation parameter ε, and a strength parameter *s* (the simple one-parameter reinforcement learning model is a special case of this three-parameter extension, with φ = 1 and ε = 0). Each parameter was fitted to maximize the log-likelihood function, separately for Proposers (φ = 0.93, ε = 0.31, s = 3.2) and Responders (φ = 0.27, ε = 0.15, s = 3.2). The fit of this reinforcement learning model against both Proposer and Responder behaviour was substantially poorer than that of our reciprocity model, according to both AIC and BIC criteria (Proposers = 2866.2 and 2883.4; and Responders = 1922.74 and 1939.9; p < 0.001). This confirmed that each player’s choices were driven largely by evaluations of utility that incorporated a reaction to their opponent’s prior behaviour.

As an exploratory analysis, we assessed whether performance of either player on the iUG was related to personality variables measured with the Action Control Scale (ACS-90)^31^; and Interpersonal Reactivity Index (IRI)^32^. Given the exploratory, post-hoc nature of these analyses, however, we do not present the results in the main body of text; instead the reader can consult them in Table S3 of the Supplementary Material. In brief, neither MAX_*Offers*_ nor MAX_*Accepts*_ were associated with scores on either personality instrument.

### Neuroimaging

**Figure 2:**
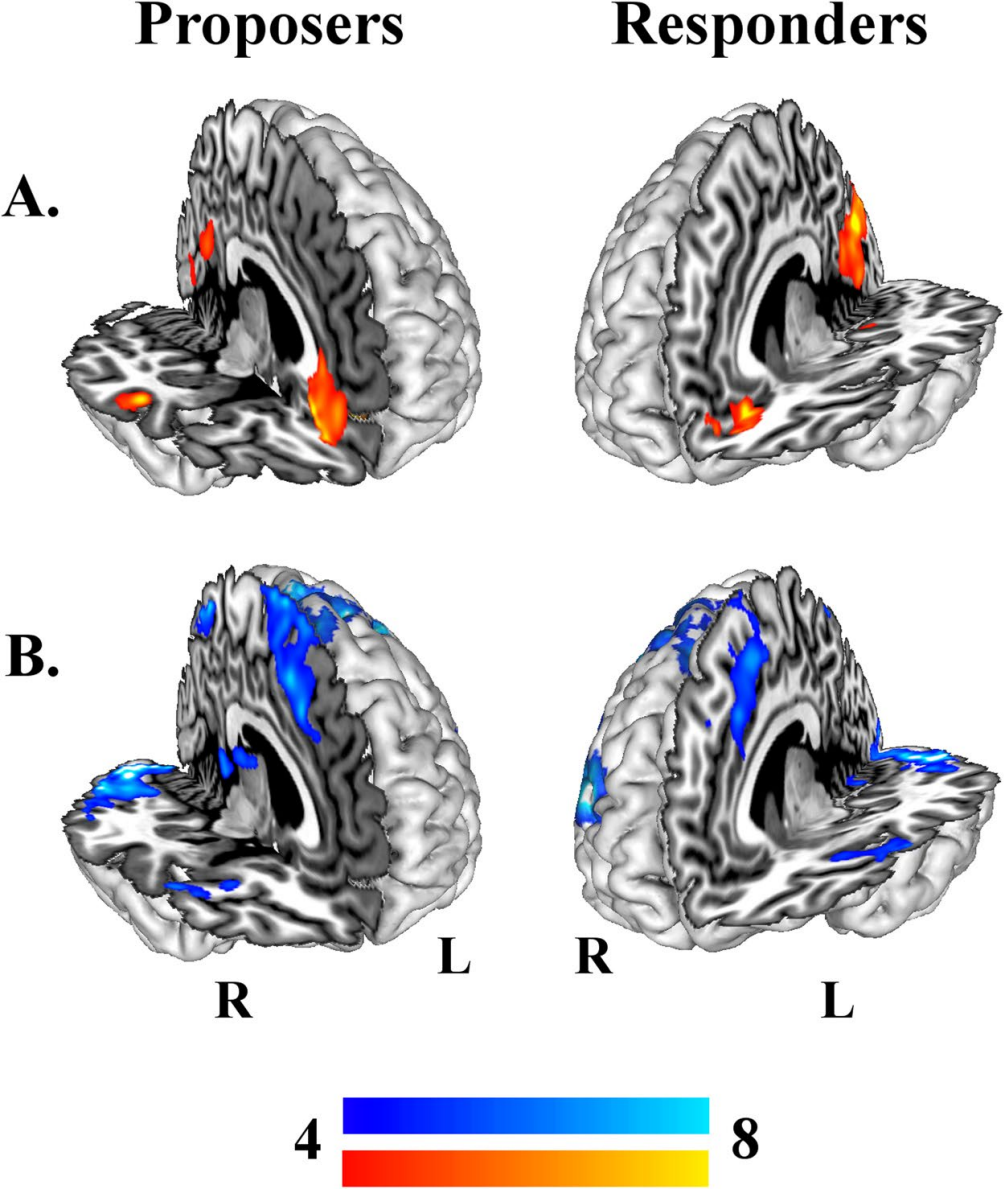
Brain responses modulated parametrically by estimates of EU. Results of the UG_*MOD*_ > CTRL contrast (p_*FWE*_ < 0.001) revealed brain regions in which BOLD response across UG rounds was modulated positively (**A**) or negatively (**B**) by EU, as estimated with our reciprocity model. Statistically equivalent patterns of EU-modulated brain responses were observed in Proposers (*left*) and Responders (*right*).

Despite the behavioural differences, the PR_*MOD*_ > PP_*MOD*_ and PP_*MOD*_ > PR_*MOD*_ contrasts revealed no significant differences in EU-modulated brain responses between the PR and PP conditions for either player. When collapsing across the two conditions, both players exhibited two similar patterns of BOLD signal expressing the UG_*MOD*_ > CTRL contrast: In the first, brain responses were modulated *positively* by EU – that is, they were greater when Proposers saw more utility in offering the least advantageously inequitable (more generous) division, and Responders saw greater utility in accepting the proposed division. This pattern encompassed primary striate and ventro-medial prefrontal cortex (vmPFC) in both players, and the superior temporal sulcus (STS) in Proposers (Fig. 2A). The second pattern represents BOLD signals modulated *negatively* by EU – these brain responses became stronger in Proposers with lower EU for the more generous division, and when Responders saw less utility in accepting the offered division. This second pattern encompassed primary and secondary striate cortices, aMCC and supplementary motor cortex (SMA), lateral prefrontal cortices and the insulae in both players; and, in Proposers, the thalamus (Fig. 2B). Clusters of brain regions exhibiting these two opposing patterns are listed in Table S4. Despite the apparent difference between players in EU modulation within the STS and thalamus, direct comparisons between player roles revealed stronger positive modulation for Proposers only in the left primary motor cortex. This was true even after more lenient thresholding (p_FWE_ < 0.01). In other words, these seemingly unique EU-modulated brain responses appear to be present in both players but to subtly (non-significantly) different degrees.

**Figure 3:**
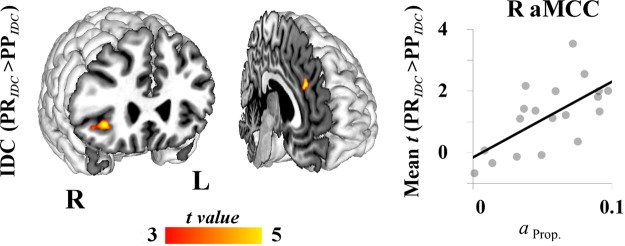
Intra-dyad correlations (IDC). Brain images illustrate greater IDC in the PR compared with the PP condition (PR_*IDC*_ > PP_*IDC*_; p_*FWE*_ < 0.05) in the right anterior insula (AI; *left*) and anterior midcingulate cortex (aMCC; *right*). The scatter plot shows that greater IDC in the PR condition was correlated positively with Proposer reciprocity estimates (α_Prop._).

As specified in Table 1, intra-dyad correlations (IDC) in brain responses measured across all rounds of each experimental condition revealed greater inter-brain alignment between interacting players on both PR and PP relative to the CTRL condition. For the PP_*IDC*_ > CTRL_*IDC*_ contrast, greater IDC was observed in bilateral occipital and extra-striate cortices, and right inferior parietal cortex. In addition to these posterior sites, the PR_*IDC*_ > CTRL_*IDC*_ contrast revealed increased IDC in lateral prefrontal cortices, aMCC, posterior cingulate cortex, and bilateral AI. The PR_*IDC*_ > PP_*IDC*_ contrast revealed the extent of this differential IDC between experimental conditions, with stronger alignment over PR compared with PP rounds in right aMCC, AI, and lateral temporal cortex, and bilateral inferior occipital cortices. To investigate whether the strength of IDC was related to the degree of reciprocity, we performed an ROI analysis at the location in which IDC expressed the PR_*IDC*_ > PP_*IDC*_ contrast maximally (aMCC; x = 4, y = 34, z = 28). This revealed that greater IDC in the PR condition within right aMCC was correlated positively with estimates of reciprocity in Proposers (ρ_[17]_ = 0.65, p = 0.003) but not in Responders (ρ_[17]_ = 0.40, p = 0.089). Interestingly, no relationships were observed between IDC expressing this contrast in the right AI (x = 34, y = 26, x = −4) and reciprocity estimates of either Proposers (ρ_[17]_ = 0.44, p = 0.062) or Responders (ρ_[17]_ = 0.26, p = 0.291). Results from the PR_*IDC*_ > PP_*IDC*_ contrast and the relationship with Proposers’ *α* estimates are illustrated in Fig. 3.

**Table 1:** Brain regions expressing intra-dyad correlations (IDC).

	Label	P_*FWE*_	Voxels	T	x	y	z	
PP_*IDC*_ > CTRL_*IDC*_	R Inferior parietal lobule	<0.001	58	5.47	32	−66	48	
	L Fusiform gyrus	<0.001	1042	7.07	−34	−80	−18	
	R Lingual gyrus	<0.001	891	7.34	20	−86	−6	
PR_*IDC*_ > CTRL_*IDC*_	L Frontal pole	0.002	61	7.22	−38	56	0	
	R aMCC	0.001	64	5.36	8	36	32	
	R Middle frontal gyrus	<0.001	159	7.51	42	32	30	
	L AI	0.001	73	5.80	−32	20	−2	
	R AI	<0.001	220	6.06	34	24	−10	
	R PCC	<0.001	518	5.66	32	−74	56	
	R Inferior parietal lobule	0.003	60	5.07	28	−64	46	
	L Middle occipital gyrus	<0.001	3044	8.27	−28	−86	2	
	R Inferior occipital gyrus	<0.001	2552	9.94	36	−86	−4	
PR_*IDC*_ > PP_*IDC*_	R aMCC	0.022	42	5.74	4	34	28	*
	R AI	<0.001	75	5.57	34	26	−4	
	R Medial temporal gyrus	0.028	40	6.33	60	−28	−12	
	L Inferior occipital gyrus	<0.001	100	5.79	−42	−80	−8	
	R Inferior occipital gyrus	<0.001	405	6.76	42	−80	−8	
	L Cerebellum	0.016	52	6.07	−26	−72	−24	

The table lists peak voxels within significant clusters expressing condition-specific intra-dyad correlations, as revealed by random-effects paired-samples t-tests for each contrast. Coordinates are specified in MNI space (2 mm^3^ resolution). P-values are given after family-wise error (FWE)-correction.

*Coordinates of the voxel around which a sphere was formed for ROI analysis (see text).

## Discussion

By scanning the brains of two individuals simultaneously while they are engaged in recursive economic exchanges with one another, we have explored brain processes associated with the bidirectional reciprocity characterising real-world, repeated dyadic interactions. This revealed three important findings: First, by modelling EU in a way that incorporates the degree of reciprocity displayed by each interactant, we show that both players’ choices on the iUG were influenced not only by their own payoff or social preferences, but also by their reactions to their opponent’s prior behaviour. Second, both players exhibited opposing patterns of neural response modulated positively or negatively by these estimates of EU. Such modulation reveals neural coupling, whereby the brain of one interactant responds to the behaviour of their interaction partner. Third, neural signals within right AI and aMCC are correlated between interacting players, particularly during exchanges that require choices between advantageous and disadvantageous inequity – those in which decisions are more likely to be driven by reciprocal tendencies. Interestingly, this pattern of inter-brain alignment was stronger with more reciprocating Proposers.

Cox *et al*.’s^24^ reciprocity model estimates EU by considering a range of individual-specific parameters. By weighing each player’s payoff against that of their opponent, it incorporates any risk aversion shown by Proposers^26^ or norm-seeking behaviour^33^ and avoidance of subjugation by Responders^5,34^. In our adaptation, however, we incorporated the additional probability that the Responder would accept an offer given their previous decisions. As such, risk aversion shown by the Proposer is modelled as a flexible adaptation to the Responder’s behaviour updated on a round-by-round basis. The reciprocity model also considers the extent to which a player’s choices are influenced by their emotional reaction to the prior behaviour of their interaction partner: If the Proposer predicts that their opponent is likely to reject the more selfish division, *and* they consider the Responder to have reacted reasonably to past offers, they see more utility in increasing their payoff at a cost to their own. Conversely, if the Proposer believes that the Responder has behaved uncooperatively in the past, they will be unwilling to change their egoistic motives despite their predictions (positive and negative reciprocity, respectively). Likewise, the Responder is more likely to accept a division that disadvantages themselves disproportionately more than their opponent if they consider the Proposer to have behaved fairly (generously, or with justified selfishness) in the past; but they see greater utility in rejecting such offers as a means of retaliating against prior unacceptable offers. Our adapted reciprocity model outperformed a self-regarding model without any estimate of reciprocity (a model that considered only inequity aversion for Responders and adaptive risk aversion for Proposers) and a reinforcement-learning model^30^.

We interpret the superior fit of our model to reflect the novel aspects of our experimental design, which allowed a more accurate simulation of real-world social decision making. In our two-choice iUG (a) repeated exchanges were made between the same two individuals, allowing both players to adapt and express their reactions to the opponent’s behaviour; (b) Responders saw the choices from which Proposers selected their offer, and Proposers saw the decisions of the Responder; and (c) two conditions were implemented to encourage stronger reciprocity between players. This differs from other paradigms in which the social context is largely removed; many studies employ a one-shot version of the UG whereby the offers on each round are made by different anonymous Proposers^28,33,35^, players’ intentionality is masked^28^, or feedback about their choices is concealed from their opponent^26^. Fairness evaluations have been shown to adapt over multiple rounds, however; Responders compare offers against normative reference points that change over successive rounds in response to Proposers’ behaviour, and both subjective feelings and affective neural responses are sensitive to violations of these adaptive norms^33,36^. Proposers also adapt to their opponent, but on a strategic level; a lower frequency of generous offers on the Dictator Game is taken as evidence that such proposals reflect strategic self interest^37^. Further, Winter and Zamir^38^ demonstrate that Proposers’ strategies emerge during the course of the game, becoming more fair or unfair in response to, respectively, unforgiving or tolerant Responders. Likewise, Billeke *et al*.^11^ report that while some Proposers adapt their offers to Responder’s behaviour, others adopt more unwavering strategies. We extend these findings by accurately modelling choices over multiple exchanges with the same known partner, permitting expressions of reciprocity during reputation building; players with low reciprocity appeared to try and maximise their advantage by establishing a “tough” reputation with unwavering unfair offers, and/or occasionally rejecting lower fair offers^39^.

Our modification of the iUG might also explain the lack of association between self-reported trait empathy and proposals or acceptances/rejections observed in our exploratory analyses. Barraza and Zak^40^, for example, report more generous proposals after empathy induction in participants high on trait empathy. This relationship between Proposer behaviour and empathy was observed in a one-shot version of the UG between anonymous players, however, a context in which reciprocal tendencies cannot influence adaptive behaviour over repeated exchanges. Similarly, Shamay-Tsoory *et al*.^41^ observed that patients with lesions to the vmPFC who reported less empathy (perspective taking) were more likely to reject offers, but these Responders played against a pre-programmed computer. Finally, Lockwood *et al*.^42^, report that reward-based learning of self- and other-reward was related to individual’s empathy, but this learning was observed in response to non-social symbolic stimuli. Over the course of our modified iUG, players had the opportunity to maximise their payoff by learning how best to adapt to the behaviour of their opponent. The fact that expressions of reciprocity were unrelated to trait empathy suggests that such adaptation reflects self-oriented strategies or affectivity, rather than other-oriented considerations (e.g., guilt). Importantly, Proposers’ offers on each round were restricted to those presented by specific choice sets, which were known to the Responder. As such, on some rounds they were free to propose selfish offers without feeling remorse.

Turning now to our neuroimaging results, both players expressed two opposing patterns of brain response modulated by EU estimates: Firstly, in the ventro-medial prefrontal cortex (vmPFC) of both players, stronger neural responses were elicited for divisions with higher EU – that is, more generous divisions for the Proposer and their acceptance by Responders. This converges with and extends existing findings; fair offers have been shown to engage the vmPFC more than unfair offers^8^, and patients with vmPFC lesions are more inclined to accept unfair offers^36^. Hutcherson *et al*.^43^ propose that vmPFC combines various information to weigh value for one’s self against that for another (player), and uses the information to generate adaptive responses. Our findings demonstrate that this brain region is implicated specifically in positive valuations. In contrast, throughout the aMCC and AI the responses of both players’ brains were stronger for divisions with lower EU – in other words, selfish divisions and offers that were rejected. The pattern of negatively modulated brain responses also advances previous findings; meta-analyses^8,9^ show consistent engagement of ACC/aMCC and AI of Responder’s brains in response to unfair offers, and neural responses in dorsal ACC and bilateral AI of Responders’ brains were found to be modulated by the degree of payoff *inequity* in unfair offers^35^. Given the accuracy of our reciprocity model in predicting players’ choices, these two patterns of EU-modulated brain responses appear to reflect opposing evaluations of utility driven by reactions to the behaviour of our interaction partner(s), which then drive opposing behavioural adaptations – specifically, positive or negative reciprocal responses.

There is accumulating evidence that the gyral aspect of the ACC processes the rewards for others, whereas the sulcus seems more sensitive to first-person reward^7,44^. Together with our findings, this indicates that the aMCC might compute the difference between self- and other-reward, whereas AI performs an emotional evaluation of any reward discrepancy that drives an affective (reciprocal) response to unfair reward discrepancy. This functional dissociation between aMCC and AI might explain the specificity of relationship between Proposer reciprocity estimates and inter-brain coupling in the aMCC during rounds that require decisions between advantageous and disadvantageous inequity; in our procedure, both players saw the choice set from which Proposers had to make decisions, and would have processed the difference in inequity presented by the two constituent divisions. In contrast, any affective reaction to the offered division would have been greater in Responders than Proposers, resulting in less covariance between players. Future studies should attempt to delineate the functions of these two brain regions during economic exchanges by modelling responses of the aMCC and/or AI as mediators of reciprocal choices – specifically, offers of unfair divisions and their rejections. Alternatively, our findings could be extended by investigating whether the parameters used in our model to estimate reciprocity – specifically, parameters representing players’ emotional state (*θ*) and choice stochasticity (*ϵ*) – serve to modulate intra-subject brain responses differentially. This might dissociate between neural processes associated with emotional reactivity and error processing during economic choices.

Brain regions encompassed by the two opposing patterns of EU-modulated responses have been implicated in different forms of learning during social decision making. The reinforcement learning framework suggests that decision making is driven by differences between predicted and actual reward outcomes (prediction errors). The ACC (particularly the sulcal aspect) is involved in reward prediction errors^45^, especially those concerning the rewards that others will receive^46^. In contrast, the lateral temporal cortex is engaged during social prediction errors^45^ and its response profile differentiates between individuals according to their social learning strategy^16^ – it is engaged more in individuals who predict rewards on the basis of their interaction partners’ behaviour, relative to those who act according to more simple learned associations between their own actions and rewards. The differential sensitivity of these two brain systems that we have observed points to a dissociable contribution of reward-based and social learning processes in utility evaluations of generous (more costly) and selfish offers during complex, repeated interactions.

The equivalence of EU-modulated brain responses observed between the PR and PP conditions suggests that players engaged in similar evaluations of utility in both types of exchange. Wang *et al*.^12^ also report similar brain responses between players in dorsal ACC. This might also explain why both players expressed highly similar patterns of EU modulation; Responders took less time to accept/reject an offer than Proposers took to make the proposal on both conditions, suggesting that Responders had already begun a similar evaluation of the choice sets before an offer was made. Moreover, both players were slower to make their choices on rounds in which choice sets involved decisions between advantageous and disadvantageous inequity, suggesting that such decisions were more cognitively demanding. Since the proportions of selfish offers and their acceptance were greater on these PR rounds, these decisions appear to involve a mutual appreciation of the intention behind selfishness. Interestingly, estimates of Proposers’ reciprocity were correlated positively with their response times and negatively with the number of selfish offers made on PR exchanges – the more adaptive they were, the less selfish their offers. Finally, greater inter-brain alignment in aMCC on PR compared with PP rounds was associated strongly with the degree of Proposers’ reciprocity. Taken together, we propose that inter-brain alignment reflects a mutual effort of players to adapt to their interaction partner by inferring the intentions behind their actions – a process that involves an evaluation of their prior behaviour. Consistent with this interpretation, the response of the aMCC has been associated with task complexity, uncertainty, predicted value, and social decision making^7,44,47^. Furthermore, hyperscanning research consistently demonstrates neural coupling within the ACC^48–50^, which is suggested to reflect accuracy in individuals’ representations of their interaction partners’ intentions^48^ and estimates of their behaviour^19,49^. Such an interpretation fits our pattern of results nicely: On PR rounds, which require decisions between advantageous and disadvantageous inequity, we observed greater inter-brain alignment within aMCC among dyads comprising more reciprocating Proposers – the player who initiates each exchange.

Our adapted iUG paradigm affords multiple applications: For example, inter-brain effects could represent effective neuromarkers for the quality of social communication^1^, providing assessment of social dimensions along which certain psychiatric illnesses might be described^51^. It is important to acknowledge the limitations of our study that can be addressed in future research, however: Firstly, we have not considered some important factors that might influence the degree of reciprocity shown by either player. Behaviour during the UG appears to be influenced partly by variability in strategic reasoning^52^ and prosocial predispositions^53^. We observed large variability in the estimates of reciprocity among our sample of Proposers that appeared to be unrelated to trait empathy, and future studies should examine if and how individual differences in other personality variables influence reciprocal behaviour during iUG. Furthermore, by examining only all-male dyads we have investigated a very specific type of dyadic interaction, and we have not explored potential sex differences in inter-brain effects^54^. Secondly, our design did not include choice sets that present a fair allocation of payoff, so it was not possible to contrast this behaviour with that following unfair options. Future studies should incorporate this condition to study all possible outcomes of repeated interaction. Finally, while our measure of brain-to-brain alignment across entire rounds permitted us to examine the degree of neural coupling through a bidirectional exchange, this crude measurement offers no insights into directionality. Neural coupling during sequential exchanges will necessarily be circular – just as the Responder’s brain synchronises to the offer of the Proposer, their decision to accept or reject will lead to systematic neural responses in the Proposer that influence future offers. To investigate such circular brain-to-brain coupling, methods for assessing directed between-brain dependencies should be developed for hyperscanning research (e.g., inter-brain psychophysiological interactions).

## Methods

### Participants

The initial sample comprised 40 males recruited from various faculties of Masaryk University, Czech Republic, who participated for monetary compensation. These individuals were paired to form 20 age-matched dyads (mean age difference = 1.2 years), the members of which had never met prior to the experiment. Male-male dyads were measured exclusively to avoid potentially confounding factors of mixed-sex interactions. Neuroimaging data from both participants comprising one dyad were omitted due to excessive head motion (see below). The 38 males comprising the remaining 19 dyads were all right-handed, had a mean age of 24.6 years (standard deviation [SD] = 3.7; range = 19.8–38.0), reported normal or corrected-to-normal vision and no history of neurological diseases or psychiatric diagnosis. All participants provided informed consent prior to the experimental procedure, which was approved by the Research Ethics Committee of Masaryk University. All methods were carried out in accordance with the declaration of Helsinki.

### Procedure

**Figure 4:**
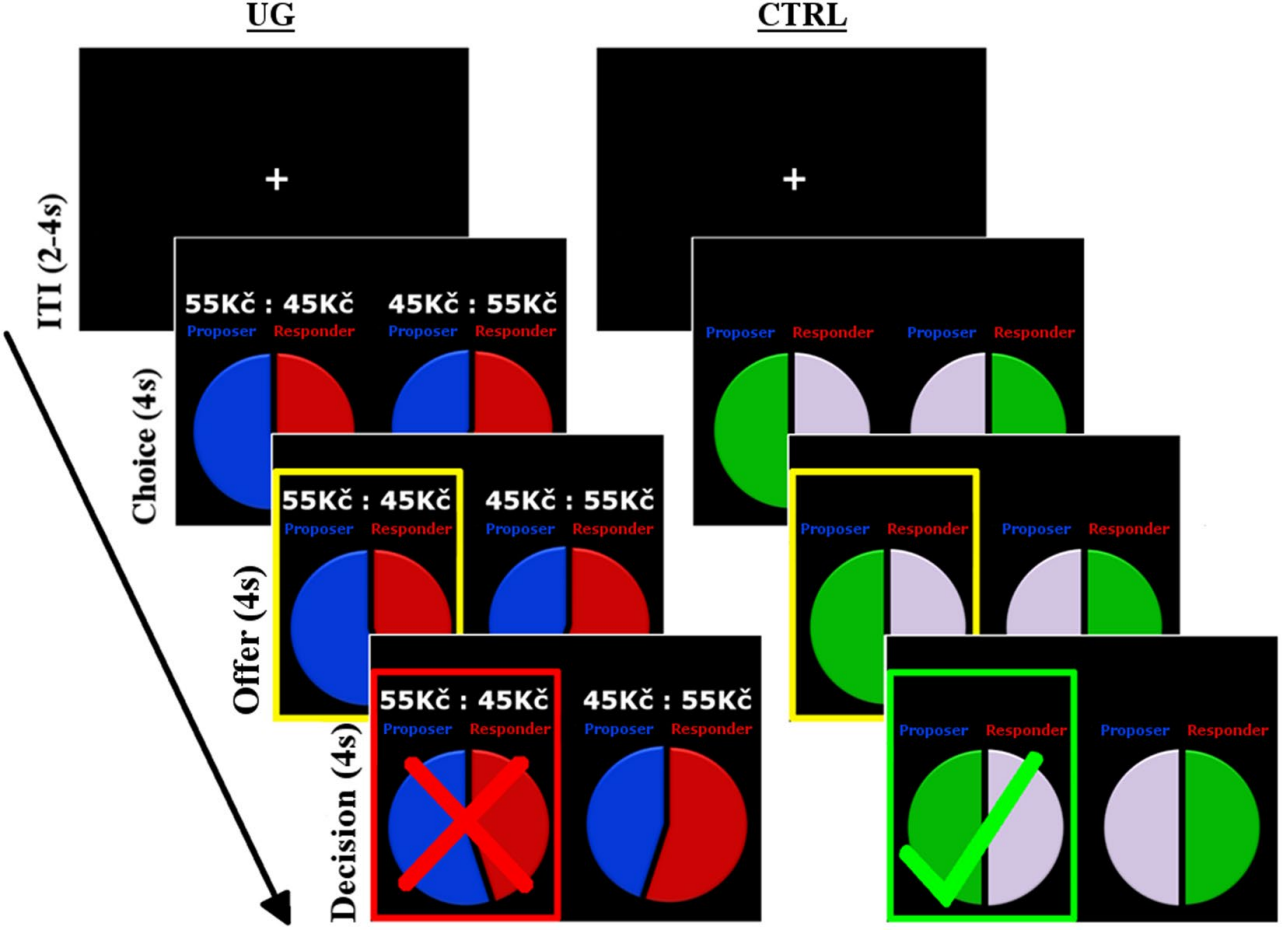
Event sequence. Timing of stimulus presentation for an example UG (*left*) and CTRL round (*right*), comprising 4-second *Choice*, *Offer* and *Decision* periods. In these examples, the offer made by the Proposer on a PR round is rejected by the Responder, while the offer made on the CTRL round is accepted.

Participants were introduced to one another for the first time on the day of scanning, during which they exchanged names and shook hands before being sent to one of two scanners located in adjacent rooms (see *Imaging Protocol*). Player roles were assigned randomly at the start but remained fixed throughout the procedure – one participant played the role of Proposer and the other Responder on all rounds. Fixing roles in this way allowed players the opportunity to learn about and adapt to their partner’s behaviour over a relatively short period. Players were told explicitly that throughout the experiment they would play with the same individual to whom they had just been introduced, and confirmed that they believed this to be true throughout the experiment. Each dyad underwent two functional runs performed successively in a single scanning session. In an event-related fashion, the two runs together comprised 120 rounds (events) of the iUG divided equally among two types of exchange (see *Stimuli*) and 60 rounds of a control condition (CTRL). Each UG round started with the Proposer being given four seconds to choose one of two divisions of the pie (the choice set; see *Stimuli*) between themselves and the Responder (*Choice* period). After this fixed period, the Proposer’s offer was highlighted for four secs (*Offer* period), during which the Responder could either accept or reject the proposal. After this four-sec period the Responder’s decision was then presented for a final four secs (*Decision* period). The exact same procedure was followed on CTRL rounds, but the choice set comprised two alternative divisions of colour between the players; rather than dividing a pie, Proposers were required to choose the colour they preferred for themselves and the colour that should go to the Responder, and the Responder then accepted or rejected that offer. Both players were instructed that CTRL rounds had no monetary consequence. Each round ended with a jittered inter-trial interval, with a fixation cross presented pseudo-randomly for 2–4 (mean = 3) secs. An example UG and CTRL round is illustrated in Fig. 4. The same fixed sequence of choice sets (one for each run) was used for all pairs, which was defined by a genetic algorithm for design optimisation^55^ set to maximise contrast detection between conditions (see below). All stimuli were presented to both players simultaneously – Responders saw the initial choice set from which Proposers selected their offer, and Proposers saw the Responder’s accept/reject decision. Players were instructed at the start that they would receive the outcome of six rounds selected at random, and the mean payout was 270 CZK (approx. €10). At no point was any information given to participants on the number of rounds remaining in the task.

As pilot data for a future study, this sample also completed two personality instruments: the ACS-90 and the IRI. Since no *a priori* hypotheses were formulated concerning these data, they are not presented in this paper. Instead, the reader is referred to the Supplementary Material for further information.

### UG Stimuli

On each round of the iUG, players were presented with a choice of two possible divisions of 100 CZK (approx. €4) that differed in the degree of inequity (the “choice set”), and Proposers were required to select one division to offer the Responder. An example choice set is illustrated in Fig. 4, and Supplementary Table S1 lists all the choice sets used in the experiment. To encourage positive and negative reciprocity, we selected 10 choice sets for which repeated proposals and acceptances of minimally inequitable divisions were lowest in behavioural piloting. The choice sets took two forms: On Proposer-Responder (PR) rounds, one division presented the Proposer with advantageous inequity while the second presented them with disadvantageous inequity – in other words, greater relative payoff was achieved by the Proposer for one division but the Responder in the other (e.g., 70:30|30:70). Conversely, on Proposer-Proposer (PP) rounds both divisions presented a greater relative payoff for the Proposer, differing only in magnitude (e.g., 70:30|60:40). Presenting a choice between advantageous and disadvantageous inequity on PR rounds was intended to encourage greater expressions of positive or negative reciprocity from both players.

### Reciprocity Model

Unlike other distributional preference models that take into account only the final relative payoff between players^34,56^, Cox *et al*.’s^24^ reciprocity model attempts to fit the behavioural observation that choices depend not only on the final monetary distribution but also on any available alternatives. Furthermore, this model also considers that player choices are influenced largely by their emotional reactions to their partner’s prior behaviour – specifically, whether their proposal or decision to accept or reject reflects positive or negative reciprocity. Finally, unlike higher beliefs equilibrium models^29,57^ the reciprocity model is tractable and enables the estimation of behavioural parameters. In our adaptation, for each player the EU of each division of the pie was specified as:

1 $$U(x,100-x)=x+(\theta \,+\,{\epsilon })(100-x)$$

here, *x* is the player’s portion of the division, *θ* is a scalar representing their emotional state, and *ϵ* represents random shock with standard logistic distribution. Random shock represents an unobserved component of the utility function – a random variable that adds stochasticity to each player’s choice behaviour (e.g., unintended responses). The emotional state was formulated as:

2 $$\theta ={\alpha }_{i}(x-{x}_{0})$$

Equation (2) incorporates a player-specific reciprocity parameter, α, which serves to weight a comparison of the player’s share, $x$, against a fairness reference point, ${x}_{0}$, by the extent to which a player’s choices are influenced by their partner’s prior behaviour. The reference point, ${x}_{0},$ is a parameter estimated with α – it is different for each choice set. Using this utility function, we modelled round-by-round EU for both players. The Responder accepts a proposal if:

3 $$x+(\theta \,+\,{\epsilon })(100-x) > 0$$

The Proposer offers the least advantageously inequitable (more generous) division if:

4 $${P}_{1}({x}_{1}+(\theta +{\epsilon })(100-{x}_{1})) > \,{P}_{2}({x}_{2}+(\theta +{\epsilon })(100-{x}_{2}))$$

In equation (4), ${x}_{1}$ and ${x}_{2}$ represent the division with minimal (or disadvantageous) and maximal advantageous inequity, respectively, and ${P}_{i}$ represents the probability that the Responder will accept a division given their prior behaviour. In other words, the Proposer makes an offer that benefits themselves maximally only if they believe the offer is likely to be accepted. The Supplementary Material gives a full description of the procedures with which the various parameters were estimated.

### Imaging Protocol

For each individual, functional and structural MR data were acquired with one of two identical 3T Siemens Prisma scanners and 64-channel bird-cage head coil. Players were allocated to one of the two scanners in a counterbalanced fashion, ensuring an even number of Proposers and Responders were scanned in each. Blood-oxygen-level dependent (BOLD) images were acquired with a *T*_2_*-weighted echo-planar imaging (EPI) sequence with parallel acquisition (i-PAT; GRAPPA acceleration factor = 2; 34 axial slices; TR/TE = 2000/35 msec; flip angle = 60°; matrix = 68 × 68 × 34, 3 × 3 × 4 mm voxels). Axial slices were acquired in interleaved order, each slice oriented parallel to a line connecting the base of the cerebellum to the base of orbitofrontal cortex permitting whole-brain coverage. Functional imaging was performed in two runs, both comprising 690 volumes (23 mins). Four dummy volumes were acquired at the beginning of each run to allow the gradients to reach steady state. For localisation and co-registration, a high-resolution *T*_1_-weighted structural MR image was acquired prior to the functional runs (MPRAGE, TR/TE = 2300/2.34 msec; flip angle = 8°; matrix = 240 × 224 × 224, 1 mm^3^ voxels). For a given dyad, volume acquisition was synchronised between scanners (mean asynchrony = 1.13 [SD = 3.83] msec) with use of a programmable signal generator (Siglent SDG1025, www.siglent.com; mean acquisition delay = 10 [SD = 3.49] msec).

### Pre-processing

For every subject, each of the two time-series were pre-processed separately using a variety of tools packaged within FMRIB’s software library (FSL^58^), full details of which are provided in the Supplementary Materials. Importantly, both players from one pair exceed our exclusion criterion of 1 mm of movement in any direction for either run, and were omitted from all subsequent analyses.

### General linear modelling

All fMRI data modeling was performed in the same platform – SPM12 (http://www.fil.ion.ucl.ac.uk). General linear modelling was performed on the pre-processed time-series in a two-step process: At the individual level, within-subject fixed-effects analyses were used for parameter estimation across both runs. Event-related responses were modelled with durations determined by the participants’ response time in each period of interest (see below), convolved with the canonical hemodynamic response function provided by SPM12: to capture brain responses that reflect reciprocal reactions in each player to their partner’s prior behaviour, for Proposers we modelled the *Choice* periods of each round until an offer was selected, while for Responders it covered the *Offer* period until a decision had been made to accept or reject the proposed division. This resulted in three task regressors for each participant, corresponding to the mean effect of the respective period in PR, PP or CTRL rounds. The remaining part(s) of the rounds were modelled as regressors of no interest. For the PR and PP task regressors we added parametric modulators that expressed the round-by-round EU estimated with the reciprocity model (PR_*MOD*_ and PP_*MOD*_); and by collapsing across the PR and PP conditions we also examined the modulatory effect throughout all UG rounds (UG_*MOD*_). To examine brain responses in Proposers and Responders separately, statistical evaluation of parameter estimates from these first-level analyses were performed in the following group-level whole-brain random-effects contrasts using one-sample t-tests: PR_*MOD*_ *vs*. PP_*MOD*_, UG_*MOD*_ > CTRL. Comparisons between players were then performed with independent-sample t-tests of the same contrasts. Cluster-wise thresholding was applied at p < 0.001, with family-wise error (FWE) correction for multiple comparisons.

### Intra-dyad Correlations

Brain-to-brain alignment on the iUG was measured with condition-specific intra-dyad correlations. To achieve reliable correlation coefficients, we combined the three phases of each round to maximise the number of volumes over which BOLD signals were correlated (6 volumes [12-sec rounds] × 60 rounds [2 runs × 30 rounds per condition]). Although this prevented us from examining alignment in each phase separately, it allowed us to capture the overall degree of alignment that occurred throughout the course of each bilateral exchange; highly correlated brain responses should be observed throughout all phases only when players share an understanding of the intentions behind each other’s actions. Brain signals should be aligned during the *Choice* phase when both players have similar evaluations of the options comprising a choice set, and should remain synchronised during the *Offer* and *Decision* phases when the intention behind a proposal and its acceptance/rejection is appreciated by both opponents.

For each Proposer-Responder dyad, using the grey-matter mask provided in SPM12 (ICBM-151) we calculated the condition-specific correlation in BOLD signal between spatially corresponding voxels of each player’s concatenated time-series, before converting correlation coefficients to z-scores using the Fischer transformation. This resulted in 19 whole-brain z-maps for each condition, with each voxel expressing the degree of condition-specific intra-dyad correlation (IDC) between a given Proposer and Responder at that spatial location (see Supplementary Fig. S1). We then compared the degree of IDC between conditions by performing the following group-level, whole-brain contrasts with random-effect paired-samples t-tests (cluster-level correction with FEW; p_*FWE*_ < 0.05): PR_*IDC*_ > CTRL_*IDC*_, PP_*IDC*_ > CTRL_*IDC*_, PR_*IDC*_ > PP_*IDC*_, and PP_*IDC*_ > PR_*IDC*_. Finally, to investigate if the relative increase in IDC on PR compared with PP rounds was related to the degree of reciprocity shown between players, we performed a region-of-interest (ROI) analysis; specifically, from each player we extracted mean t-values from a sphere of 5 mm radius centred on the voxel expressing the PR_*IDC*_ > PP_*IDC*_ contrast maximally, and performed non-parametric Spearman correlations to assess the relationship between these values and player reciprocity estimates (*α*).

### Data Availability

The datasets generated and analysed during the current study are available from the corresponding author on reasonable request.

## Electronic supplementary material

Supplementary Materials
