# Supplementary material for: A dual-fMRI investigation of the iterated Ultimatum Game reveals that reciprocal behaviour is associated with neural alignment
Source: Sci Rep. 2018 Jul 18;8:10896. doi: 10.1038/s41598-018-29233-9 (PMC6051991; doi:10.1038/s41598-018-29233-9)
Supplement: Supplementary file 1 — Supplementary Materials [file 41598_2018_29233_MOESM1_ESM.docx]

**Title:** A dual-fMRI investigation of the iterated Ultimatum Game reveals that reciprocal behaviour is associated with neural alignment.

**Authors:** Daniel J. Shaw*^1,2^, Kristína Czekóová^2^, Rostislav Staněk^3^, Radek Mareček^2^, Tomáš Urbánek^4^, Jiří Špalek^3,^ Lenka Kopečková^3^, Jan Řezáč^3^ & Milan Brázdil^2^

***Reciprocity Model***

As we assume that *ϵ* has a logistic distribution, the probability of the Responder accepting an offer can be expressed as the value of the cumulative distribution function of the logistic distribution:

1. $F\left( z \right)=e^{z}\left( e^{z}+1 \right)^{-1}$ $F\left( z \right)=e^{z}\left( e^{z}+1 \right)^{-1}$

This is evaluated at:

1. $z=x/{(100-x)}+\alpha_{i} x-\alpha_{i}x_{0}$ $z=x/{(100-x)}+\alpha_{i} x-\alpha_{i}x_{0}$

Responders’ α and $x_{0}$ parameters are estimated by maximizing the log-likelihood function:

1. $\ln L=\sum_{i} \sum_{j} y_{ij}\ln F\left( z \right)+\left( 1-y_{ij} \right)\ln\left( 1-F\left( z \right) \right)$

Variable $y_{ij}$ is binary variable, taking a value of one if Responder *i* in round *j* accepted the offer and zero otherwise. For Proposers, after some algebraic manipulations it can be shown that the probability of offering the division with minimal advantageous inequity (MIN offers) is given by the value of the cumulative distribution function of the logistic distribution $F\left( v \right)$:

1. $v=\frac{P_{1}x_{1}-P_{2}x_{2}}{P_{1}\left( 100-x_{1} \right)-P_{2}\left( 100-x_{2} \right)}+\frac{\alpha_{i}(P_{1}x_{1}\left( 100-x_{1} \right)-P_{2}x_{2}\left( 100-x_{2} \right))}{P_{1}\left( 100-x_{1} \right)-P_{2}\left( 100-x_{2} \right)}-\alpha_{i}x_{0}$

Estimation of the Proposer’s α and $x_{0}$ parameters is complicated by the fact that the probability of making the MIN offer depends upon the expected decision of the Responder. To estimate the expected probability of acceptance on a given round, we considered the Responder’s previous behaviour; specifically, using the procedure described above we estimate the Responder’s parameters α and $x_{0}$ using only the last *M* rounds (the *memory* parameter). The memory parameter therefore represents the range of preceding Responder choices over which the Proposer’s prediction of their opponent’s upcoming response is maximised. The estimated value of parameters α and $x_{0}$ determine the probability that the Responder will accept a particular offer via the relationship $P_{i}=F(z)$. Given these probabilities of acceptance, the Proposer’s parameters α and $x_{0}$ are estimated by maximizing the log-likelihood function:

1. $\ln L=\sum_{i} \sum_{j} y_{ij}\ln F\left( v \right)+\left( 1-y_{ij} \right)\ln\left( 1-F\left( v \right) \right)$

Again, $y_{ij}$ is a binary variable taking a value of one if the Proposer *i* in period *j* makes the more generous offer and zero otherwise. The value of *M* was based upon the fit to the actual Proposer’s behaviour; namely, acceptance probabilities were first estimated on the basis of the Responder’s decisions over all possible ranges of preceding UG rounds (2-119), and the Proposer’s α and $x_{0}$ parameters were then re-estimated with every possible range *M*. The optimal range was defined as the best-fitting model by log-likelihood. The *memory* parameter was estimated only for Proposers because their payoff on a given round depends upon the expected (unknown) decision of the Responder. In contrast, the round-by-round payoff for the Responder depends upon the Proposer’s offer, which is known.

***Nested models***

The first nested model assumes that α=0 for both Proposers and Responders. As such, the utility function becomes:

1. $U\left( x,100-x \right)=x+\varepsilon(100-x)$

The Responder accepts an offer if:

1. $x+\varepsilon\left( 100-x \right)>0$

Given this condition, we calculated acceptance probabilities, $P_{i}$, as the value of logistic distribution at the point $x/(100-x)$. The proposer makes the least advantageously inequitable (more generous) offer if:

1. $P_{1}\left( x_{1}+\varepsilon(100-x_{1}) \right)> P_{2}(x_{2}+\varepsilon\left( 100-x_{2} \right))$

In equation 8, $x_{1}$ and $x_{2}$ represents the options with minimal and maximal advantageous inequity, respectively, and $P_{i}$ represents the probability that the Responder will accept given offer.

The second nested model assumes that α=0 only for Proposers, and we apply our reciprocity model for Responders – acceptance probabilities, $P_{i}$, remain the same. Again, the proposer makes the more or less generous offer based on the condition:

1. $P_{1}\left( x_{1}+\varepsilon(100-x_{1}) \right)> P_{2}(x_{2}+\varepsilon\left( 100-x_{2} \right))$.

We refer to these as self-regarding models because, although the utility function includes the other player’s monetary payoff (100-x), the mean of $\varepsilon$ is zero and at this point the player does not care about the monetary payoff of their opponent.

***Reinforcement learning model***

The strategy space of the Proposer is to make a generous or selfish offer, and for the Responder it is to accept or reject a proposal. The reinforcement learning model consists of initial propensities, a reward function, a rule for updating propensities, and a probabilistic choice rule. We assume that initial propensities, *q(1)*, are equal for both strategies:

1. $q\left( 1 \right)=sX$

In the above equation, the strength of the initial propensity is given by parameter *s,* and *X* is the average payoff in the game. The reward function, *R(x)*, is equal to the monetary payoff on a given round; that is, $R\left( x \right)=x$. The rule for updating propensities for strategy *a* is:

1. $q_{a}\left( t+1 | s\left( t \right)=a \right)=\left( 1-\varphi\right)q_{a}\left( t \right)+(1-\varepsilon)R(x)$
2. $q_{a}\left( t+1 | s\left( t \right)\neq a \right)=\left( 1-\varphi\right)q_{a}\left( t \right)+\varepsilon R(x)$

Here, $\varepsilon$ is the experimentation parameter and $\varphi$ is the forgetting parameter. The probabilistic choice rule gives the probability that strategy *a* will be played:

1. $p_{a}\left( t \right)=\frac{s_{a}(t)}{\sum s(t)}$

The parameters were fitted to maximize the log-likelihood function. The behavior of Proposers and Responders were modeled separately, that is, model parameters were different for each player.

***Pre-processing of Neuroimaging Data***

Initial motion correction was performed with MCFLIRT, using the middle volume of the run as a reference. Two subjects exceed our exclusion criterion of 1 mm of movement in any direction for either run, and were omitted from all subsequent analyses. Slice-time correction was achieved with Fourier-space time-series phase-shifting. Using FEAT v6.00, each functional image was high-pass filtered across time to remove low-frequency drifts (Gaussian-weighted least-squares straight line fitting; sigma = 60 sec), and spatially smoothed using a 5 mm full-width half-maximum Gaussian kernel. The time-series were intensity normalised using grand-mean scaling of the entire 4D dataset by a single multiplicative factor, minimising any unspecific time effects. To identify any signal related to noise sources (e.g., residual motion, physiological noise) we performed a probabilistic independent component analysis using MELODIC (Beckmann, 2012), decomposing the time-series into 50 independent spatial and temporal components. Artefactual components were identified automatically with the Spatially Organized Component Klassifikator (SOCK; Bhaganagarapu, Jackson & Abbott, 2013), and signal relating to these noise components was regressed out of the time-series with *fsl_regfilt*. Using FLIRT these pre-processed time-series were then registered linearly to the corresponding anatomical image using boundary-based registration (Greve & Fischl, 2009), the anatomical image was registered linearly to the MNI-152 template (12 DOF), and the time-series were registered linearly to MNI space (9 DOF) by concatenating both transformation matrices.

| **Condition** | **Choice Sets** | **Payoff Difference** |
| --- | --- | --- |
| **PP** | 70:30\|55:45 | 15 |
|  | 70:30\|60:40 | 10 |
|  | 65:35\|55:45 | 10 |
|  | 65:35\|60:40 | 5 |
|  | 60:40\|55:45 | 5 |
| **PR** | 70:30\|30:70 | 40 |
|  | 65:35\|35:65 | 30 |
|  | 65:35\|45:55 | 20 |
|  | 60:40\|40:60 | 20 |
|  | 55:45\|45:55 | 10 |

Table S1. Choice sets. Underlined values represent the payoff to the Proposer in each of the two constituent options, and the leftmost option presents the division with maximal advantageous inequity (MAX). The difference in each player’s payoff between the MAX division and the alternative option is shown beside each choice set. Each of the 10 choice sets was presented 12 times throughout the experiment, with the MAX division on the left or right for an equal number of rounds.

| Memory (*M*) parameter | Proposer EU | Reciprocity (*α*) parameter | Prediction accuracy (%) |
| --- | --- | --- | --- |
| 20 | .89 | .98 | .94 |
| 30 | .92 | .98 | .96 |
| 40 | .94 | .98 | .97 |
| 50 | .96 | .99 | .97 |
| 60 | .98 | 1.00 | .97 |
| 80 | .99 | 1.00 | .98 |

Table S2. The degree of similarity across reciprocity models with different *Memory* parameters. Values present the Spearman correlation coefficient between models with different *Memory* parameters and that with the optimal value (73) for round-by-round estimates of Proposer EU, Proposers’ reciprocity parameters, and the accuracy of model predictions for Proposers’ choices.

***Associations between personality and iUG performance***

As an exploratory investigation, we investigated the relationship between personality variables and the performance of Proposers and Responders on the iUG. Personality was assessed via two self-report instruments: The Action Control Scale (ACS-90^31^) and the Interpersonal Reactivity Index (IRI^32^). The ACS-90 is a 36-item questionnaire that measures an individual’s ability to regulate affective states quickly and flexibly in response to environmental demands (action orientation) rather than fixating on them in a change-preventing volitional mode (state orientation). It consists of three sub-scales: *Action orientation after failure* vs. preoccupation (AOF); *Prospective and demand-related action orientation* vs. hesitation (AOD); and *Action orientation during activity performance* vs. volatility (AOP). Since AOP is considered less relevant than AOF and AOD to personality theory^31^, we focused on the latter two dimensions. The IRI is a 28-item multi-dimensional questionnaire of individual differences in trait empathy. It consists of four seven-item subscales that measure discrete empathic tendencies: *Perspective Taking* ([PT] adopting spontaneously the psychological perspective of others), *Fantasy Scale* ([FS] transposing oneself imaginatively into the feelings and actions of fictitious characters), *Empathic Concern* ([EC] adopting "other-oriented" feelings of sympathy and concern), and *Personal Distress* ([PD] having “self-oriented" feelings of personal anxiety and unease in tense interpersonal settings). The subscales of PT and FS index cognitive empathy, while those on EC and PD reflect affective empathy. As shown in Table S3, while certain subscales of these instruments were correlated with one another they were related only to Responders’ response time (RT) in the PP condition. Given the exploratory nature of these analyses we do not present them in the main body of text, but we discuss their potential implications in the Discussion.

|  |  |  | α | AOF | AOD | PT | FS | EC | PD | MAX*_Offers_*/*_Accepts_* | | RT | | IDC | |
| --- | --- | --- | --- | --- | --- | --- | --- | --- | --- | --- | --- | --- | --- | --- | --- |
|  |  |  |  |  |  |  |  |  |  | PP | PR | PP | PR | AI | aMCC |
| Proposers |  | α | 1 |  |  |  |  |  |  |  |  |  |  |  |  |
|  |  | AOF | -.30 | 1 |  |  |  |  |  |  |  |  |  |  |  |
|  |  | AOD | -.22 | .49* | 1 |  |  |  |  |  |  |  |  |  |  |
|  |  | PT | -.05 | .27 | .18 | 1 |  |  |  |  |  |  |  |  |  |
|  |  | FS | .07 | -.09 | -.17 | .03 | 1 |  |  |  |  |  |  |  |  |
|  |  | EC | -.02 | -.31 | -.13 | .32 | .29 | 1 |  |  |  |  |  |  |  |
|  |  | PD | -.17 | -.49* | -.15 | -.31 | .49* | .44 | 1 |  |  |  |  |  |  |
|  | MAX  *_Offers_*/*_Accepts_* | PP | -.88** | .26 | .13 | -.12 | .01 | .01 | .22 | 1 |  |  |  |  |  |
|  |  | PR | -.78** | .19 | .25 | -.24 | -.13 | .02 | .19 | .82** | 1 |  |  |  |  |
|  | RT | PP | -.22 | .21 | -.11 | -.27 | -.20 | -.18 | .10 | .42 | .33 | 1 |  |  |  |
|  |  | PR | .58** | .02 | -.27 | .03 | -.12 | -.34 | -.26 | -.47* | -.60** | .32 | 1 |  |  |
|  | IDC | AI | .44 | .14 | .04 | .13 | .17 | -.15 | -.23 | -.37 | -.34 | .13 | .66** | 1 |  |
|  |  | aMCC | .65** | .01 | -.25 | -.20 | .03 | -.42 | -.28 | -.43 | -.44 | .11 | .58** | .48* | 1 |
|  |  |  |  |  |  |  |  |  |  |  |  |  |  |  |  |
| Responders |  | α | 1 |  |  |  |  |  |  |  |  |  |  |  |  |
|  |  | AOF | .01 | 1 |  |  |  |  |  |  |  |  |  |  |  |
|  |  | AOD | .18 | .51* | 1 |  |  |  |  |  |  |  |  |  |  |
|  |  | PT | -.12 | -.08 | -.26 | 1 |  |  |  |  |  |  |  |  |  |
|  |  | FS | -.01 | -.63** | -.41 | -.03 | 1 |  |  |  |  |  |  |  |  |
|  |  | EC | .03 | .06 | -.27 | .42 | 0.31 | 1 |  |  |  |  |  |  |  |
|  |  | PD | -.06 | -.71** | -.38 | .07 | .63** | .06 | 1 |  |  |  |  |  |  |
|  | MAX  *_Offers_*/*_Accepts_* | PP | -.07 | -.18 | -.13 | .01 | -.06 | -.06 | .22 | 1 |  |  |  |  |  |
|  |  | PR | -.15 | -.07 | .01 | -.03 | -.17 | -.06 | .11 | .82** | 1 |  |  |  |  |
|  | RT | PP | -.09 | -.59** | -.21 | .11 | .54* | .22 | .42 | -.29 | -.33 | 1 |  |  |  |
|  |  | PR | .09 | -.32 | -.09 | .26 | .24 | -.01 | .04 | -.66** | -.73** | .63** | 1 |  |  |
|  | IDC | AI | -.26 | .11 | -.08 | -.24 | .17 | -.08 | -.15 | .27 | .23 | -.33 | -.42 | 1 |  |
|  |  | aMCC | -.40 | .31 | .40 | -.22 | -.23 | -.15 | -.19 | -.02 | .03 | -.24 | -.25 | .48* | 1 |

Table S3. Spearman correlations among measures of personality and performance on the iterated Ultimatum Game.

|  |  |  | **Proposers** | | | | | **Responders** | | | | |
| --- | --- | --- | --- | --- | --- | --- | --- | --- | --- | --- | --- | --- |
|  | **Label** |  | **Voxels** | **t** | **x** | **y** | **z** | **Voxels** | **t** | **x** | **y** | **z** |
| **Positive Modulation** | Frontal Pole | R | 22 | 5.65 | 36 | 44 | 28 | 305 | 9.54 | 33 | 53 | 25 |
|  | IFG | R | 30 | 7.15 | 51 | 5 | 28 |  |  |  |  |  |
|  | MFG | L |  |  |  |  |  | 36 | 6.66 | -27 | 32 | 25 |
|  | SFG | R | 73 | 7.43 | 33 | -1 | 64 |  |  |  |  |  |
|  | Putamen | R |  |  |  |  |  | 22 | 6.36 | 15 | 8 | -2 |
|  | Thalamus | L | 81 | 7.35 | -12 | -28 | 7 |  |  |  |  |  |
|  |  | R |  |  |  |  |  | 68 | 7.38 | 12 | -19 | 4 |
|  | Mid Insula | L | 424 | 8.47 | -39 | -1 | 4 | 444 | 9.36 | -42 | -4 | 13 |
|  |  | R |  |  |  |  |  | 296 | 10.52 | 42 | -4 | 10 |
|  | Precentral gyrus | L | 892 | 10.06 | -54 | -25 | 49 |  |  |  |  |  |
|  | PPC | R |  |  |  |  |  | 187 | 10.18 | 27 | -61 | 37 |
|  | SMG | L |  |  |  |  |  | 828 | 10.45 | -60 | -25 | 40 |
|  | ITS | R |  |  |  |  |  | 55 | 5.35 | 54 | -28 | -17 |
|  | STS | R | 177 | 10.78 | 54 | 17 | -8 |  |  |  |  |  |
|  | IOG | L |  |  |  |  |  | 633 | 9.87 | -39 | -82 | -11 |
|  |  | R | 892 | 9.13 | 30 | -82 | -14 |  |  |  |  |  |
|  | Cerebellum | L | 113 | 11.65 | -36 | -55 | -32 |  |  |  |  |  |
|  |  | R | 41 | 6.75 | 18 | -70 | -47 | 29 | 6.18 | 48 | -58 | -29 |
| **Negative Modulation** | vmPFC | L | 520 | 9.68 | -6 | 41 | -8 | 200 | 8.09 | -6 | 38 | -11 |
|  | IPL | L | 78 | 7.85 | -45 | -76 | 40 |  |  |  |  |  |
|  |  | R | 66 | 7.38 | 51 | -58 | 16 |  |  |  |  |  |
|  | MTG | L | 47 | 8.16 | -57 | -1 | -20 |  |  |  |  |  |
|  | STS | L | 164 | 6.69 | -54 | -16 | -5 |  |  |  |  |  |
|  |  | R | 142 | 7.44 | 51 | -4 | -17 |  |  |  |  |  |
|  |  |  | 94 | 6.85 | 57 | -22 | 4 |  |  |  |  |  |
|  | Primary striate | L | 110 | 6.94 | -3 | -61 | 22 | 1697 | 10.36 | -6 | -76 | 28 |
|  |  |  | 1114 | 6.94 | -15 | -76 | -8 |  |  |  |  |  |

Table S4. Brain regions exhibiting EU-modulated BOLD responses. The table lists foci of voxel clusters expressing the UG*_MOD_*>CTRL contrast, for both Proposers (*left*) and Responders (*right*). Brain responses modulated positively or negatively by estimates of EU are presented in the top or bottom half of the table, respectively. Coordinates are specified in MNI space (2mm^3^ resolution). All clusters are significant at p*_FEW_*<.001. *Abbreviations*: I/M/SFG = inferior/middle/superior frontal gyrus, PPC = posterior parietal cortex, SMG = supramarginal gyrus, I/M/STS = inferior/middle/superior temporal gyrus, IOG = inferior occipital gyrus, vmPFC = ventro-medial prefrontal cortex, IPL = inferior parietal lobule.


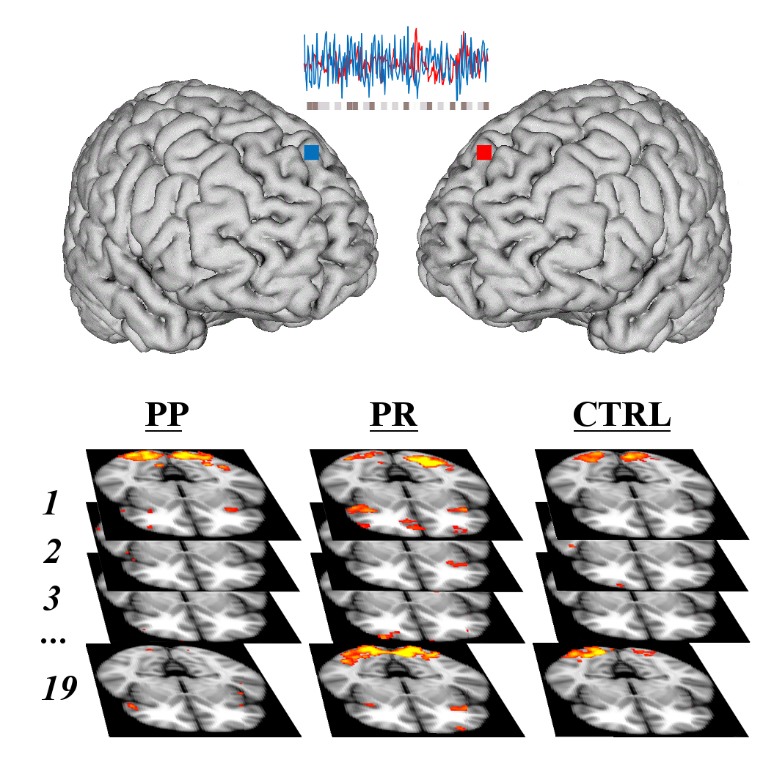


Figure S1. Sequence of IDC calculation. *Top*: Each player’s time series was first segmented into rounds and all segments relating to a given condition were concatenated. Then, for each concatenated time series the BOLD signal was extracted from pairs of spatial corresponding voxels between interacting players’ brains and correlated. *Bottom*: Correlation coefficients for each voxel were transformed into z-scores to produce 19 whole-brain IDC maps for each condition, and conditions were compared with random effects paired-samples t-tests.
